# The role of compatible solutes in desiccation resistance of *Acinetobacter baumannii*

**DOI:** 10.1002/mbo3.740

**Published:** 2018-10-02

**Authors:** Sabine Zeidler, Volker Müller

**Affiliations:** ^1^ Department of Molecular Microbiology & Bioenergetics Institute of Molecular Biosciences Goethe‐University Frankfurt am Main Frankfurt Germany

**Keywords:** *Acinetobacter baumannii*, compatible solutes, desiccation, mannitol, osmotic stress, trehalose

## Abstract

*Acinetobacter baumannii* is a nosocomial pathogen which can persist in the hospital environment not only due to the acquirement of multiple antibiotic resistances, but also because of its exceptional resistance against disinfectants and desiccation. A suitable desiccation assay was established in which *A. baumannii* ATCC 19606^T^ survived for ca. 1 month. The growth medium slightly influenced survival after subsequent desiccation. A significant effect could be attributed to the growth phase in which bacteria were dried: In exponential phase, cells were much more desiccation sensitive. The main focus of the present study was the elucidation of the role of compatible solutes, which are known to protect many bacteria under low water activity conditions, in desiccation survival of *A. baumannii*. Exogenous trehalose was shown to efficiently protect *A. baumannii* on dry surfaces, in contrast to other compatible solutes tested such as mannitol or glycine betaine. To analyze the importance of intracellularly accumulated solutes, a double mutant lacking biosynthesis pathways for mannitol and trehalose was generated. This mutant accumulated glutamate as sole solute in the presence of high NaCl concentrations and showed severe growth defects under osmotic stress conditions. However, no effect on desiccation tolerance could be seen, neither when cells were dried in water nor in the presence of NaCl.

## 1 INTRODUCTION

The opportunistic human pathogen *Acinetobacter baumannii* is a steadily rising threat in healthcare facilities worldwide, emphasized once more in 2017 by the WHO, which set carbapenem‐resistant *A. baumannii* on top of their priority list for pathogens for which research and development of new antibiotics is urgently needed (World Health Organization, 2017). The emergence of *A. baumannii* as an important nosocomial pathogen is multifactorial. Its metabolic versatility and resistances to various environmental stresses not only allow this pathogen to survive for extended periods in hospital settings, but in concert with a number of true virulence factors, afford the bacterium the ability to adapt to and successfully infect the host (Antunes, Imperi, Carattoli, & Visca, 2011; Dijkshoorn, Nemec, & Seifert, 2007; Roca, Espinal, Vila‐Farrés, & Vila, 2012; Weber, Harding, & Feldman, 2016). In particular, remarkable is the high desiccation resistance which is unusual for a Gram‐negative bacterium as survival on dry, inanimate surfaces for months or even years has been reported (Antunes et al., 2011; Jawad, Heritage, Snelling, Gascoyne‐Binzi, & Hawkey, 1996; Jawad, Seifert, Snelling, Heritage, & Hawkey, 1998; Wendt, Dietze, Dietz, & Ruden, 1997). This promotes persistence and spread in healthcare facilities. It has been reported that *A. baumannii* can not only persist for weeks on various parts of the human body (Dijkshoorn, van Vianen, Degener, & Michel, 1987), but it has also has been isolated from various places in hospitals during outbreaks, for example from furniture, door knobs, or equipment (van den Broek et al., 2006) and can survive in desiccated infant formula for 2 years (Juma, Manning, & Forsythe, 2016).

To date, few factors contributing to this extraordinary desiccation resistance are known. Besides the fact that biofilm forming strains survive longer on dry surfaces (Chiang et al., 2017; Espinal, Martí, & Vila, 2012; Orsinger‐Jacobsen et al., 2013), RecA (a protein involved in DNA repair) (Aranda et al., 2011) as well as the acylation of lipid A (Boll et al., 2015) have been reported to be involved in desiccation resistance. A proteomics study performed by Gayoso et al. (2014) revealed mainly general features associated with desiccation resistance, such as the downregulation of genes involved in transcription, translation, and cell division, and the upregulation of genes for efflux pumps and antimicrobial resistance. Combined with observed changes in membrane composition, the authors propose a so‐called “bust‐and‐boom” strategy.

In the present study, we aimed to investigate a possible role of compatible solutes in desiccation resistance of *A. baumannii*. Compatible solutes are small, organic molecules which can be accumulated in the cell in up to molar concentrations without interfering with the central metabolism (Kempf & Bremer, 1998; Roeßler & Müller, 2001). They not only provide protection from osmotic stress by counterbalancing the osmolarity, but also by stabilizing membranes and proteins. Due to these stabilizing properties, compatible solutes can protect from many other environmental stresses, including desiccation. Besides that, the beginning of drought stress is usually accompanied by an additional osmotic stress, as the concentration of soluble substances increases when the liquid evaporates (Potts, 1994). Indeed, transcriptomic analyses under desiccation conditions in several bacteria, for example *Anabaena*,* Rhodococcus,* or *Salmonella*, revealed the upregulation of genes involved in biosynthesis or transport of compatible solutes (Katoh, Asthana, & Ohmori, 2004; Leblanc, Gonçalves, & Mohn, 2008; Li, Bhaskara, Megalis, & Tortorello, 2012). In other organisms such as the cyanobacterium *Nostoc* or *E. coli*, the compatible solute trehalose is accumulated in response to drought stress (Sakamoto et al., 2009; Zhang & Yan, 2012).

In response to salt stress, *A. baumannii* accumulates glutamate, mannitol, and trehalose or, if present, takes up glycine betaine from the environment (Zeidler et al., 2017), but so far nothing is known about a possible involvement of these solutes in desiccation tolerance. In particular, trehalose is a very potent protector against desiccation used, amongst others, by anhydrobiotes (Crowe, Oliver, & Tablin, 2002), and the unusual solute mannitol, which is a radical scavenger, could be involved in protection against oxidative stress that occurs upon rehydration (Efiuvwevwere, Gorris, Smid, & Kets, 1999). Herein, we have addressed the role of compatible solutes in desiccation resistance of *A. baumannii*.

## 2 MATERIALS AND METHODS

### 2.1 Bacterial strains and culture conditions

All bacterial strains used in this study are given in Table 1. *Acinetobacter baumannii* strain ATCC 19606^T^, *Escherichia coli* DH5α, and *Bacillus subtilis* JH642 were grown at 37°C and 130 rpm, while growth conditions for *Micrococcus luteus* were 30°C and 130 rpm. Growth media were Luria Bertani broth (LB) (Bertani, 1951) or a mineral medium consisting of different mineral salts (1 g/L NH_4_Cl, 580 mg/L MgSO_4_ × 7 H_2_O, 100 mg/L KNO_3_, 67 mg/L CaCl_2_ × 2 H_2_O, 2 mg/L (NH_4_)_6_Mo_7_O_24_ × 4 H_2_O), 1 ml of the trace element solution SL9 (12.8 g/L nitrilotriacetic acid (titriplex), 2 g/L FeSO_4_ × 7 H_2_O, 190 mg/L CoCl_2_ × 6 H_2_O, 122 mg/L MnCl_2_ × 4 H_2_O, 70 mg/L ZnCl_2_, 36 mg/L MoNa_2_O_4_ × 2 H_2_O, 24 mg/L NiCl_2_ × 6 H_2_O, 6 mg/L H_3_BO_3_, 2 mg/L CuCl_2_ × 2 H_2_O, modified after Tschech and Pfennig (1984)), 20 mM sodium succinate as a carbon source, and 50 mM phosphate buffer. Stock solutions of all components were autoclaved separately. For growth under osmotic stress conditions, NaCl was added to the medium in the concentrations indicated (200–500 mM). Growth rates were determined using the “exponential growth equation” analysis of GraphPad Prism for the exponential growth phase.

**Table 1:** Bacterial strains used

Strain	Reference
*Escherichia coli* DH5α	Invitrogen™, USA
*Bacillus subtilis* JH642	BGSC, USA
*Micrococcus luteus*	DSMZ, Germany
*Acinetobacter baumannii* ATCC 19606^T^	ATCC, USA
*Acinetobacter baumannii* ATCC 19606^T^ Δ*otsB*	Zeidler et al. (2017)
*E. coli* DH5α with pBIISK_sacB/kanR_mtlD‐updown	Zeidler et al. (2018)
*Acinetobacter baumannii* ATCC 19606^T^ Δ*mtlD‐otsB*	This study

Note

BGSC: *Bacillus* Genetic Stock Center; DSMZ: Deutsche Sammlung von Mikroorganismen und Zellkulturen; ATCC: American Type Culture Collection.

### 2.2 Desiccation assays

Bacteria were grown in 5 ml cultures overnight and harvested in stationary phase, unless stated otherwise. 1 ml was harvested and washed twice in the same volume of H_2_O, with the addition of salt or compatible solutes where indicated. The same liquid was used to adjust the sample to an OD_600_ of 2.0 ± 0.1, which corresponds to 1.2 × 10^7^–3.2 × 10^9^ colony forming units (CFU) per ml. Aliquots of 20 μl of sample were applied to small polycarbonate filters (Nuclepore Track‐Etch Membrane, 13 mm, 0.4 μm), which had been sterilized by autoclaving. Where indicated, saline (0.9% NaCl), 200 mM NaCl, or 10 mM of different compatible solutes were used for washing and resuspending instead of H_2_O. The membrane filters were put in petri dishes and incubated with slightly opened lids in a climate chamber, to ensure controlled drying conditions. After defined periods at 22°C and 31% relative humidity (RH), two membrane filters were analyzed for each time point (technical duplicates). Each filter was put in a 15‐ml falcon tube containing 1 ml of sterile saline and vortexed vigorously for 30 s. Vortexing was repeated after a 30 min incubation at 37°C and 300 rpm to remove all bacteria from the filter. Appropriate dilutions were prepared in saline and 100 μl thereof plated on LB agar plates, which were then incubated at 37°C. When the remaining number of viable cells was very low, the suspension was centrifuged and the whole sample was plated in a smaller volume. Colonies were enumerated after ca. 1 day to determine the CFU per filter. Longer incubation times did not increase the number of colonies. Percent survival was determined in relation to the initial CFU value (time point 0 before incubation of the membrane filters).

To test for statistical significance, unpaired t tests were performed at defined time points. P‐values were calculated using the software GraphPad Prism. Statistical significance was assigned when *p* < 0.05.

### 2.3 Markerless mutagenesis

A double deletion mutant of the genes *mtlD* (HMPREF0010_00722, encoding a mannitol dehydrogenase) and *otsB* (HMPREF0010_01306, encoding a trehalose‐phosphate‐phosphatase) in *A. baumannii* ATCC 19606^T^ was created. A markerless Δ*otsB* mutant described before (Zeidler et al., 2017) was used to additionally delete *mtlD*. This was done by double homologous recombination as described by Zeidler et al. (2018) using the plasmid pBIISK_sacB/kanR_mtlD‐updown and the primers listed there, leading to the mutant strain *A. baumannii* ATCC 19606^T^ Δ*mtlD‐otsB*. Deletion of *mtlD* was confirmed by sequencing of the PCR product obtained with the primers mtlD_ctr_up and mtlD_ctr_down.

### 2.4 Extraction and quantification of solutes

Mannitol, trehalose, and glutamate were extracted from cells and quantified as described earlier (Zeidler et al., 2017). Briefly, bacteria were harvested in late exponential growth phase and lyophilized, followed by extraction of intracellular solutes with methanol and chloroform by a modified Bligh‐and‐Dyer method (Bligh & Dyer, 1959; Galinski & Herzog, 1990). Mannitol was determined via HPLC using a ligand exchange column (HyperREZ XP Carbohydrate Ca^2+^, Thermo Scientific) and a refractive index detector. The enzymatic test kits K‐TREH and K‐GLUT (Megazyme, Bray, Ireland) were used for quantification of trehalose and glutamate, respectively. For the protein content of freeze‐dried cells, the mean value obtained by Zeidler et al. (2017) was applied.

## 3 RESULTS

### 3.1 Establishment of a desiccation assay

**Figure 1:**
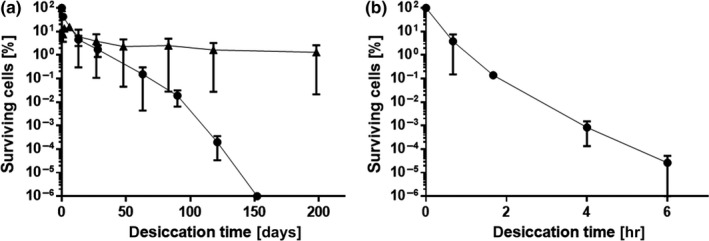
Desiccation survival of different bacterial species. Overnight cultures of bacteria grown in LB medium were washed and adjusted to OD_600_ 2.0 in H_2_O. A 20 μl aliquot of each standardized cell suspension was applied to polycarbonate membrane filters to achieve initial viable cell densities ranging from 2 × 10^6^ to 1.6 × 10^7^ CFU per filter (100%) which were then stored under desiccation (31% RH) at 22°C. Surviving cells were enumerated at designated time points. Gram‐positive bacteria: *Bacillus subtilis* JH642 (▲), *Micrococcus luteus* (●) (a). Gram‐negative bacterium: *E. coli* DH5α (b). For each experiment, mean values of at least two biological replicates are shown. Error bars represent the standard error of the mean (*SEM*)

**Figure 2:**
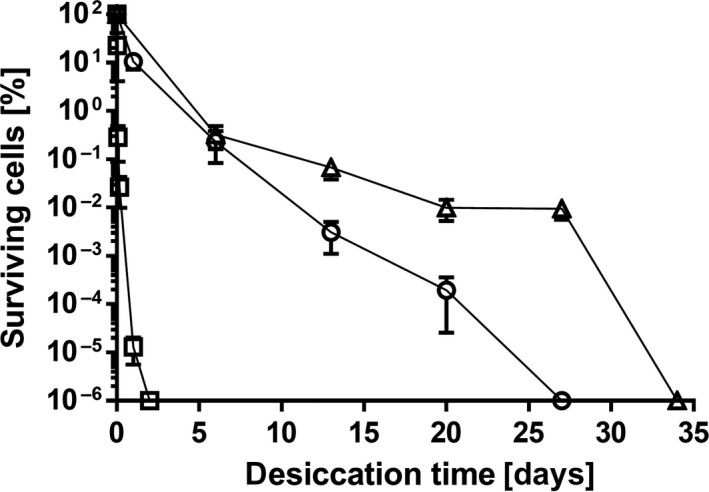
Desiccation resistance of *Acinetobacter baumannii* ATCC 19606^T^ as affected by growth medium and growth phase. Cultures were grown overnight in LB (Δ) or mineral medium (○) or to mid‐log phase (OD 0.45–0.65) in mineral medium (□), and then washed and adjusted to OD_600_ 2.0 in H_2_O. A 20 μl aliquot of each standardized cell suspension was applied to polycarbonate membrane filters to achieve initial viable cell densities ranging from 4 × 10^6^ to 4.6 × 10^7^ CFU per filter which were then stored under desiccation (31% RH) at 22°C. For each experiment, mean values out of at least four biological replicates are shown. Error bars represent the standard error of the mean (*SEM*)

In order to establish a desiccation assay suitable to investigate *A. baumannii* desiccation tolerance, we initially compared the survival of *A. baumannii* ATCC 19606^T^ with other bacteria. Bacteria were grown in LB medium overnight and washed before drying to remove all nutrients. In most desiccation assays described in literature, either water or saline is used to resuspend bacteria prior to drying. We decided to use water as was done for example in the study by Jawad et al. (1996) in order not to impose an additional salt stress on the cells. As temperature and humidity significantly influence survival, all desiccation experiments were performed in a climate chamber at 22°C and 31 % relative humidity (RH), mimicking potential physiological conditions *A. baumannii* could encounter when drying on a surface in a hospital. The assays for all bacteria in this study used starting cell suspensions standardized to an OD_600_ of 2.0, which resulted in initial viable cell densities in the range of 1.2 × 10^7^–3.2 × 10^9^ CFU/ml. This corresponded to levels of 2.4 × 10^5^–6.4 × 10^7^ CFU per membrane filter. The different bacteria tested showed a wide range of survival times, proving functionality of the assay. As expected, the spore forming *Bacillus subtilis* survived longest, followed by the other Gram‐positive organism, *Micrococcus luteus* (Figure 1a). Colonies of *M. luteus* could be detected after up to 4 months of drying, whereas *A. baumannii* ATCC 19606^T^ survived for 1 month (Figure 2), which is considerably longer than the times reported for many other Gram‐negative organisms. The common *E. coli* laboratory strain DH5α displayed high sensitivity to desiccation as viable cells could no longer be detected after only a few hours (Figure 1b).

### 3.2 Desiccation resistance of *A. baumannii* depending on growth conditions

Many factors can contribute to bacterial desiccation tolerance. To analyze the influence of the growth medium, *A. baumannii* was grown in mineral medium instead of LB. This resulted in a slight decrease in survival rate (Figure 2), which could be due to protective substances present in LB which can be taken up during growth. Desiccation resistance was therefore tested after growth in mineral medium supplemented with 1 mM of the compatible solute glycine betaine, which is contained in LB, but this did not increase survival compared to growth in non‐supplemented mineral medium (data not shown). Another factor influencing bacterial physiology is growth temperature. However, bacteria grown in mineral medium at room temperature (22°C) did not exhibit a different desiccation tolerance (data not shown). Although growth temperature and growth media did not induce appreciable changes in the desiccation resistance of *A. baumannii*, the growth phase from which bacteria were harvested was found to significantly (*p* = 0.036 after 1 day) impact survivability of cells exposed to desiccation. In comparison with stationary phase cells, exponential cells of the pathogen proved to be extremely sensitive to the stress. For example, viable stationary phase cells were still detectable after ca. 3 weeks of drying while nearly no viable exponential phase cells could be detected after just 1 day of desiccation (Figure 2).

### 3.3 Desiccation of *A. baumannii* in the presence of compatible solutes

**Figure 3:**
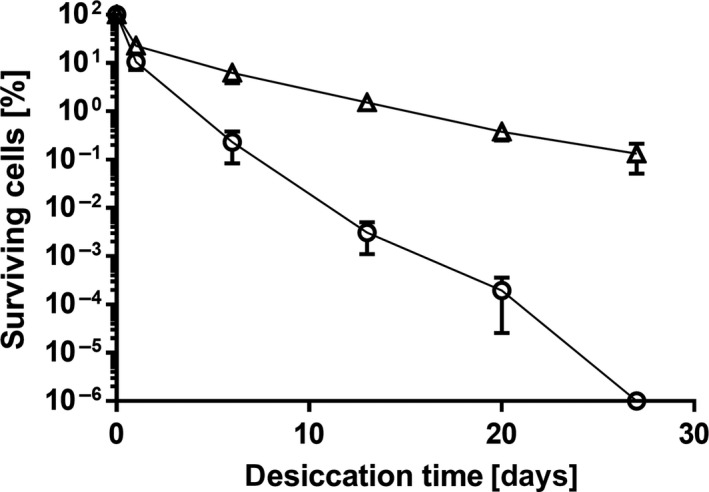
Effect of trehalose on desiccation of *Acinetobacter baumannii*. Overnight cultures of *A. baumannii* grown in mineral medium were washed and adjusted to OD_600_ 2.0 in H_2_O (○) or in 10 mM trehalose (Δ). A 20 μl aliquot of each standardized cell suspension was applied to polycarbonate membrane filters to achieve initial viable cell densities ranging from 4 × 10^6^ to 4.6 × 10^7^ CFU per filter which were then stored under desiccation (31% RH) at 22°C. For each experiment, mean values of at least four biological replicates are shown. Error bars represent the standard error of the mean (*SEM*)

Trehalose is known as a very potent desiccation protector (Billi & Potts, 2002; Crowe, Crowe, & Chapman, 1984; Elbein, Pan, Pastuszak, & Carroll, 2003). To check whether it can increase *A. baumannii* desiccation resistance, cells grown to stationary phase in mineral medium were dried in the presence of 10 mM trehalose (Figure 3). After as little as 6 days of desiccation, cells suspended in the presence of 10 mM trehalose displayed a clear advantage over those suspended only in water, as only 0.2% survivors could be detected in the case of the latter while exposure to the solute resulted in greater than 5% survivors (*p* = 0.0003). The positive effect of exogenous trehalose was even more pronounced after longer desiccation times. The rate of decrease for CFUs was significantly slower compared to that of cells suspended just in water prior to drying (*p* = 0.003 after 20 days), as 1% of these cells remained viable after 4 weeks of desiccation.

**Figure 4:**
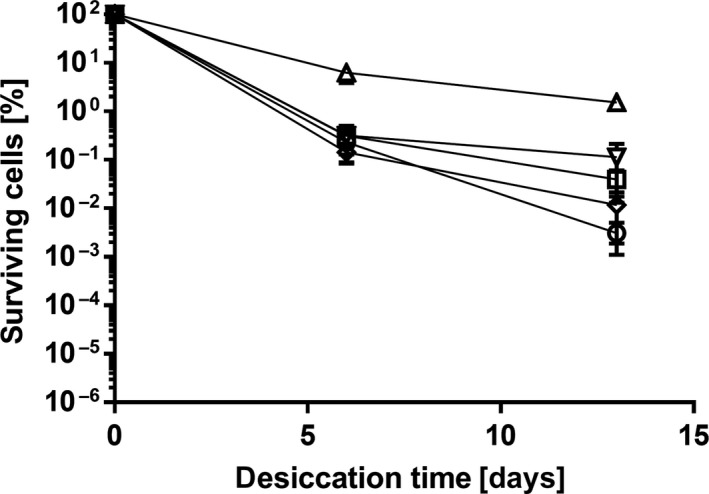
Effect of different compatible solutes on desiccation survival of *Acinetobacter baumannii*. Cultures of *A. baumannii* were grown overnight and subsequently washed and adjusted to OD_600_ 2.0 in H_2_O (○) or 10 mM of the following compatible solutes: trehalose (Δ), glutamate (□), mannitol (▽), glycine betaine (◊). Percent survival in relation to the starting CFU values (2.4 × 10^5^–4.6 × 10^7^ CFU per filter) was determined after 6 and 13 days. For each experiment, mean values of at least four biological replicates are shown. Error bars represent the standard error of the mean (*SEM*)

To analyze whether this effect is specific for trehalose or if all compatible solutes have a positive effect on desiccation survival, the experiment was repeated with 10 mM mannitol, glutamate, or glycine betaine (Figure 4). After 6 days, the survival rates in the presence of theses solutes were in the range of drying in water, thus lower than with trehalose. After 13 days, a minimal protective effect could be observed for glutamate (*p* = 0.014) but not for mannitol (*p* > 0.05), and results for glycine betaine were inconclusive due to inconsistencies in data amongst trials. However, none of these solutes led to survival rates as high as trehalose. This experiment demonstrated that not all compatible solutes have a positive effect on desiccation and that trehalose was the most effective of the tested solutes for protection of *A. baumannii* on dry surfaces.

### 3.4 Salt stress and desiccation resistance of Δ*mtlD‐otsB*

**Figure 5:**
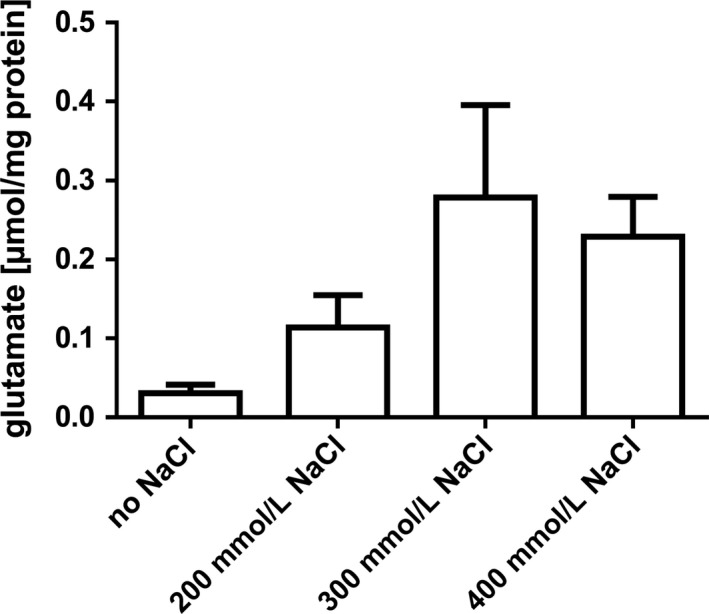
Compatible solutes in a Δ*mtlD‐otsB* mutant of *Acinetobacter baumannii*. Bacteria were grown in mineral medium with different NaCl concentrations and harvested in late exponential growth phase for determination of the intracellular solute pool. Solutes were extracted with chloroform and methanol, glutamate was quantified enzymatically. Shown are the mean values of three biological replicates, error bars represent the standard deviation

**Figure 6:**
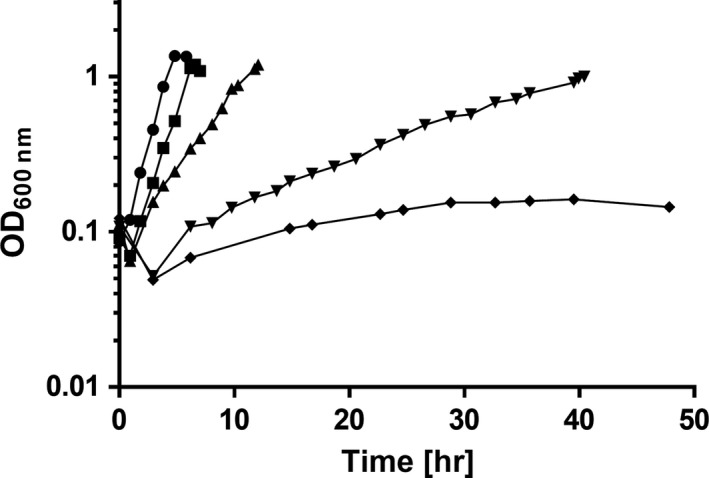
Effect of NaCl on growth of *Acinetobacter baumannii* Δ*mtlD‐otsB*. Cells were grown in mineral medium (●) or in mineral medium with the addition of 200 (■), 300 (▲), 400 (▼), or 500 mM NaCl (♦). One representative experiment out of three independent biological replicates is shown

Many bacteria are known to produce compatible solutes in response to drought stress. To elucidate whether intracellularly accumulated solutes are needed for survival of *A. baumannii* on dry surfaces, we established a deletion mutant defective in the biosynthesis pathways for both mannitol and trehalose. As expected, this mutant did produce glutamate as sole compatible solute during growth at high salinities (200–400 mM NaCl). Up to 0.3 μmol glutamate/mg of protein were accumulated, which is slightly less as determined earlier for the wild type (Figure 5) (Zeidler et al., 2017). Growth of the Δ*mtlD‐otsB* double mutant was significantly impaired at high NaCl concentrations, comparable to the Δ*mtlD* single mutant described in Zeidler et al. (2018) (Figure 6). Without additional NaCl, the growth rate of the mutant was 0.60 ± 0.04 hr^−1^, which is nearly identical to the wild type (92%) (Zeidler et al., 2017). In the mineral medium supplemented with 200, 300, or 400 mM NaCl, growth rates were 0.50 ± 0.04 hr^−1^ (86% of the wild type), 0.34 ± 0.08 (67%), and 0.06 ± 0.01 (19%), respectively, and at 500 mM NaCl no growth was observed. The addition of glycine betaine restored growth in the presence of 500 mM NaCl to a level comparable to non‐osmotic stress conditions (data not shown).

**Figure 7:**
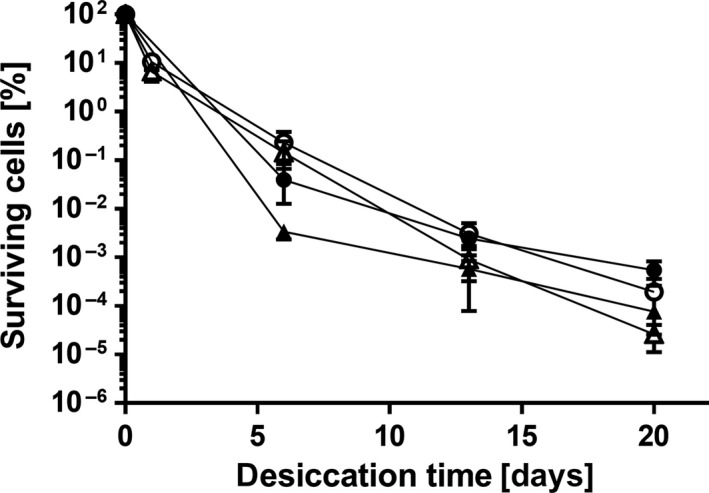
Desiccation resistance of *Acinetobacter baumannii* Δ*mtlD‐otsB*. Wild type (○, ●) and the markerless deletion mutant Δ*mtlD‐otsB* (Δ, ▲) were grown overnight in mineral medium and washed and dried in H_2_O (empty symbols, starting value 2‐6.4 × 10^7^ CFU per filter) or grown in mineral medium supplied with 200 mM NaCl, followed by washing and drying in 200 mM NaCl (filled symbols, starting value 6.4 × 10^6^–1.9 × 10^7^ CFU per filter). For each experiment, mean values of at least four biological replicates are shown. Error bars represent the standard error of the mean (*SEM*)

However, when the mutant was grown in mineral medium and dried in water, survival rates were similar to the wild type (Figure 7), indicating that production of the compatible solutes mannitol and trehalose is not beneficial under these conditions. We hypothesized that the solutes could be required when bacteria were dried in a moderate salt concentration, as evaporation of the water increases the concentration, which might lead to the need for protective solutes as in other bacteria (Beblo‐Vranesevic, Galinski, Rachel, Huber, & Rettberg, 2017; Bonaterra, Camps, & Montesinos, 2005; Reina‐Bueno et al., 2012; Welsh & Herbert, 1999). Yet, drying in saline instead of water did not affect dying of the wild type nor of the mutant (data not shown). We assumed that the drying time might be too short to effectively induce production and accumulation of solutes. Therefore, in a further experiment both strains were grown in mineral medium containing 200 mM NaCl, a condition inducing accumulation of mannitol and trehalose in *A. baumannii*, and subsequently dried in the presence of the same salt concentration (Figure 7). Neither wild type nor the double mutant showed altered survival in the presence of salt.

## 4 DISCUSSION

Desiccation is one of the most important and severe stress factors that terrestrial bacteria must overcome in order to survive (Ramos et al., 2001). Desiccation tolerance enables the persistence of foodborne pathogens and plays a major role in hospital outbreaks, for example of *Burkholderia cepacia* or *A. baumannii* (Beuchat et al., 2013; Drabick, Gracely, Heidecker, & Lipuma, 1996; Jawad et al., 1998). Desiccation and subsequent rehydration lead, amongst others, to denaturation of proteins and DNA damage (Potts, 2001). Simultaneously, microorganisms are frequently exposed to oxidative and osmotic stress as well as nutrient starvation, and, therefore, the respective stress responses overlap (Ramos et al., 2001). One ubiquitous key element in protection from various stress conditions is the so‐called compatible solutes (Welsh, 2000). We previously analyzed compatible solutes and their role in osmotic stress protection of *A. baumannii* (Zeidler et al., 2017, 2018) and now aimed to elucidate a possible role in desiccation resistance.

In our study, *A. baumannii* ATCC 19606^T^ survived for approximately 1 month on dry surfaces, which is longer than was reported for the same strain in an older study by Jawad et al. (1996) but similar to that in another more recent study (Giannouli et al., 2013). When comparing survival times with data of other studies, it must be considered that many factors influence desiccation tolerance, for example the surface used and the inoculum volume (Hanczvikkel & Tóth, 2018; Neely, 2000; Wendt et al., 1997). In addition, it is highly strain‐specific: clinical strains of *A. baumannii* exhibit a significantly higher desiccation resistance than laboratory strains (Giannouli et al., 2013; Jawad et al., 1998). The same holds true for other organisms; for *E. coli*, the reported survival times range from a few hours to several months (Kramer, Schwebke, & Kampf, 2006). Nevertheless, it is agreed that in general, Gram‐positive bacteria have a higher desiccation resistance than Gram‐negative (Janning & In't Veld, 1994; Nocker, Fernández, Montijn, & Schuren, 2012), which was also reflected in our assay.

To date, only a few studies have investigated the desiccation resistance of *A. baumannii*, and to our knowledge, we are the first to report on the sensitivity of the exponential phase cells of this pathogen to drying. Although this is a newly reported feature for *A. baumannii*, similar traits have been reported in other bacteria such as *Salmonella enterica* (Gruzdev, Pinto, & Sela‐Saldinger, 2012) or *Sinorhizobium meliloti* (Vriezen, de Bruijn, & Nüsslein, 2006). This can be attributed to the fact that stationary phase cells in general are more stress resistant (Kolter, Siegele, & Tormo, 1993). Stationary phase cells of *A. baumannii* have increased resistance against oxidative stress and it has been shown that various proteins involved in stress protection are upregulated (Soares et al., 2010). In a study by Jacobs et al. (2012), the transcriptomic analysis of stationary cells of *A. baumannii* revealed the upregulation of certain genes involved in trehalose biosynthesis, and this was more pronounced in clinical strains. The authors speculated a role for trehalose in desiccation resistance.

To test the hypothesis of an involvement of trehalose or compatible solutes in general in desiccation resistance of *A. baumannii*, we first analyzed the effect of extracellular solutes. Indeed, trehalose was the only solute which significantly increased survival times. The same has been reported for other bacteria such as *E. coli* (Louis, Trüper, & Galinski, 1994), *Staphylococcus aureus* (Chaibenjawong & Foster, 2011), and *S. enterica* (Gruzdev, Pinto, et al., 2012). In all cases, glycine betaine did not have a positive effect, which is in accordance with our results. The outstanding effect of trehalose on desiccation resistance is attributed to its special chemical properties (Crowe et al., 1984; Elbein et al., 2003; Potts, 2001).

Despite the positive effect of exogenous trehalose on *A. baumannii* survival, a double mutant lacking biosynthesis genes for trehalose (*otsB*) and mannitol (*mtlD*) did not exhibit decreased survival. We assumed that trehalose accumulation could be important for persistence under dry conditions not only because the *otsBA* promoter in *A. baumannii* is activated under osmotic and temperature stress (Zeidler et al., 2017) and *otsB* is important for persistence in *Galleria mellonella* larvae (Gebhardt et al., 2015), but also because endogenous solutes are involved in desiccation tolerance of many organisms, with trehalose playing an outstanding role (Argüelles, 2000; Elbein et al., 2003; Potts, 1994). In *S. enterica*, the genes for trehalose biosynthesis (*otsBA*) are upregulated 11‐fold during desiccation (Finn et al., 2013). Transcriptomics in *Bradyrhizobium japonicum, Anabaena,* and *Rhodococcus jostii* revealed upregulation of biosynthesis pathways of compatible solutes as a common feature (Cytryn et al., 2007; Katoh et al., 2004; Leblanc et al., 2008). Also *E. coli* produces more trehalose, proline, and glutamine when dried (Zhang & Yan, 2012), and the high desiccation tolerance of *C. sakazakii* could be attributed to trehalose accumulation (Breeuwer, Lardeau, Peterz, & Joosten, 2003). A mutant strain of *Rhizobium etli* unable to synthesize trehalose exhibited a lower desiccation survival (Reina‐Bueno et al., 2012). However, this was not observed in our studies with *A. baumannii*.

Taken together, our data clearly show enhanced desiccation survival for cells in stationary phase and a protective role of exogenous trehalose, but do not point toward a connection between accumulation of trehalose or mannitol and desiccation resistance. Still it should be kept in mind that the exact experimental conditions can influence the results to a great extent (Finn et al., 2013), reflected for example by the fact that Gruzdev, McClelland, et al. (2012) could not detect upregulation of solute transporters in *Salmonella*, in contrast to others studying the same organism (Finn et al., 2013; Li et al., 2012). Therefore, the fact that a proteomics analysis in *A. baumannii* did not reveal upregulation of proteins involved in synthesis of compatible solutes (Gayoso et al., 2014) does not definitely exclude a possible connection. Whether the effect of exogenous trehalose is physiologically relevant remains unclear. Just recently the intensified use of trehalose as a food additive has received negative publicity as it is associated with the increasing threat of *Clostridium difficile* as a nosocomial pathogen (Collins et al., 2018). It is important to know that trehalose in food could help *A. baumannii* to persist, especially as this pathogen has already been detected in several foods (Dijkshoorn et al., 2007).

## CONFLICT OF INTEREST

The authors declare no conflict of interest.

## AUTHOR CONTRIBUTION

SZ and VM designed the research, analyzed the data, and wrote the manuscript. SZ performed the experiments. All authors read and approved the final manuscript.

## ETHICS STATEMENT

This research did not involve studies with human or animal subjects, materials or data; therefore, no ethics approval is required.

## Data Availability

All data are included in the main manuscript. Raw data and materials are available on request.

## References

[mbo3740-bib-0001] Antunes, L. C. S. , Imperi, F. , Carattoli, A. , & Visca, P. (2011). Deciphering the multifactorial nature of *Acinetobacter baumannii* pathogenicity. PLoS ONE, 6, e22674 10.1371/journal.pone.0022674 21829642 PMC3148234

[mbo3740-bib-0002] Aranda, J. , Bardina, C. , Beceiro, A. , Rumbo, S. , Cabral, M. P. , Barbé, J. , & Bou, G. (2011). *Acinetobacter baumannii* RecA protein in repair of DNA damage, antimicrobial resistance, general stress response, and virulence. Journal of Bacteriology, 193, 3740–3747. 10.1128/JB.00389-11 21642465 PMC3147500

[mbo3740-bib-0003] Argüelles, J. C. (2000). Physiological roles of trehalose in bacteria and yeasts: A comparative analysis. Archives of Microbiology, 174, 217–224. 11081789 10.1007/s002030000192

[mbo3740-bib-0004] Beblo‐Vranesevic, K. , Galinski, E. A. , Rachel, R. , Huber, H. , & Rettberg, P. (2017). Influence of osmotic stress on desiccation and irradiation tolerance of (hyper)‐thermophilic microorganisms. Archives of Microbiology, 199, 17–28. 10.1007/s00203-016-1269-6 27443666

[mbo3740-bib-0005] Bertani, G. (1951). Studies on lysogenesis. 1. The mode of phage liberation by lysogenic *Escherichia coli* . Journal of Bacteriology, 62, 293–300. 14888646 10.1128/jb.62.3.293-300.1951 PMC386127

[mbo3740-bib-0006] Beuchat, L. R. , Komitopoulou, E. , Beckers, H. , Betts, R. P. , Bourdichon, F. , Fanning, S. , … ter Kuile, B. H. (2013). Low‐water activity foods: increased concern as vehicles of foodborne pathogens. Journal of Food Protection, 76, 150–172. 10.4315/0362-028X.JFP-12-211 23317872

[mbo3740-bib-0007] Billi, D. , & Potts, M. (2002). Life and death of dried prokaryotes. Research in Microbiology, 153, 7–12. 10.1016/S0923-2508(01)01279-7 11881900

[mbo3740-bib-0008] Bligh, E. G. , & Dyer, W. J. (1959). A rapid method of total lipid extraction and purification. Canadian Journal of Biochemistry and Physiology, 37, 911–917. 10.1139/y59-099 13671378

[mbo3740-bib-0009] Boll, J. M. , Tucker, A. T. , Klein, D. R. , Beltran, A. M. , Brodbelt, J. S. , Davies, B. W. , & Trent, M. S. (2015). Reinforcing lipid A acylation on the cell surface of *Acinetobacter baumannii* promotes cationic antimicrobial peptide resistance and desiccation survival. MBio, 6, e00478–15. 25991684 10.1128/mBio.00478-15 PMC4442142

[mbo3740-bib-0010] Bonaterra, A. , Camps, J. , & Montesinos, E. (2005). Osmotically induced trehalose and glycine betaine accumulation improves tolerance to desiccation, survival and efficacy of the postharvest biocontrol agent *Pantoea agglomerans* EPS125. Fems Microbiology Letters, 250, 1–8. 10.1016/j.femsle.2005.06.028 16002241

[mbo3740-bib-0011] Breeuwer, P. , Lardeau, A. , Peterz, M. , & Joosten, H. M. (2003). Desiccation and heat tolerance of *Enterobacter sakazakii* . Journal of Applied Microbiology, 95, 967–973. 10.1046/j.1365-2672.2003.02067.x 14633024

[mbo3740-bib-0012] Chaibenjawong, P. , & Foster, S. J. (2011). Desiccation tolerance in *Staphylococcus aureus* . Archives of Microbiology, 193, 125–135. 10.1007/s00203-010-0653-x 21088825

[mbo3740-bib-0013] Chiang, S. R. , Jung, F. , Tang, H. J. , Chen, C. H. , Chen, C. C. , Chou, H. Y. , & Chuang, Y. C. (2017). Desiccation and ethanol resistances of multidrug resistant *Acinetobacter baumannii* embedded in biofilm: The favorable antiseptic efficacy of combination chlorhexidine gluconate and ethanol. Journal of Microbiology, Immunology and Infection, 10.1016/j.jmii.2017.02.003 28732564

[mbo3740-bib-0014] Collins, J. , Robinson, C. , Danhof, H. , Knetsch, C. W. , van Leeuwen, H. C. , Lawley, T. D. , … Britton, R. A. (2018). Dietary trehalose enhances virulence of epidemic *Clostridium difficile* . Nature, 553, 291–294. 10.1038/nature25178 29310122 PMC5984069

[mbo3740-bib-0015] Crowe, J. H. , Crowe, L. M. , & Chapman, D. (1984). Preservation of membranes in anhydrobiotic organisms ‐ the role of trehalose. Science, 223, 701–703. 10.1126/science.223.4637.701 17841031

[mbo3740-bib-0016] Crowe, J. H. , Oliver, A. E. , & Tablin, F. (2002). Is there a single biochemical adaptation to anhydrobiosis? Integrative and Comparative Biology, 42, 497–503. 10.1093/icb/42.3.497 21708744

[mbo3740-bib-0017] Cytryn, E. J. , Sangurdekar, D. P. , Streeter, J. G. , Franck, W. L. , Chang, W.‐S. , Stacey, G. , … Sadowsky, M. J. (2007). Transcriptional and physiological responses of *Bradyrhizobium japonicum* to desiccation‐induced stress. Journal of Bacteriology, 189, 6751–6762. 10.1128/JB.00533-07 17660288 PMC2045231

[mbo3740-bib-0018] Dijkshoorn, L. , Nemec, A. , & Seifert, H. (2007). An increasing threat in hospitals: Multidrug‐resistant *Acinetobacter baumannii* . Nature Reviews: Microbiology, 5, 939–951. 18007677 10.1038/nrmicro1789

[mbo3740-bib-0019] Dijkshoorn, L. , van Vianen, W. , Degener, J. E. , & Michel, M. F. (1987). Typing of *Acinetobacter calcoaceticus* strains isolated from hospital patients by cell‐envelope protein profiles. Epidemiology and Infection, 99, 659–667. 10.1017/S0950268800066516 3428372 PMC2249253

[mbo3740-bib-0020] Drabick, J. A. , Gracely, E. J. , Heidecker, G. J. , & Lipuma, J. J. (1996). Survival of *Burkholderia cepacia* on environmental surfaces. Journal of Hospital Infection, 32, 267–276. 10.1016/S0195-6701(96)90037-7 8744511

[mbo3740-bib-0021] Efiuvwevwere, B. J. O. , Gorris, L. G. M. , Smid, E. J. , & Kets, E. P. W. (1999). Mannitol‐enhanced survival of *Lactococcus lactis* subjected to drying. Applied Microbiology and Biotechnology, 51, 100–104. 10.1007/s002530051369

[mbo3740-bib-0022] Elbein, A. D. , Pan, Y. T. , Pastuszak, I. , & Carroll, D. (2003). New insights on trehalose: A multifunctional molecule. Glycobiology, 13, 17R–27R. 10.1093/glycob/cwg047 12626396

[mbo3740-bib-0023] Espinal, P. , Martí, S. , & Vila, J. (2012). Effect of biofilm formation on the survival of *Acinetobacter baumannii* on dry surfaces. Journal of Hospital Infection, 80, 56–60. 10.1016/j.jhin.2011.08.013 21975219

[mbo3740-bib-0024] Finn, S. , Händler, K. , Condell, O. , Colgan, A. , Cooney, S. , McClure, P. , … Fanning, S. (2013). ProP is required for the survival of desiccated *Salmonella enterica* serovar Typhimurium cells on a stainless steel surface. Applied and Environmental Microbiology, 79, 4376–4384. 10.1128/AEM.00515-13 23666329 PMC3697505

[mbo3740-bib-0025] Galinski, E. A. , & Herzog, R. M. (1990). The role of trehalose as a substitute for nitrogen‐containing compatible solutes (*Ectothiorhodospira halochloris*). Archives of Microbiology, 153, 607–613. 10.1007/BF00245273

[mbo3740-bib-0026] Gayoso, C. M. , Mateos, J. , Méndez, J. A. , Fernández‐Puente, P. , Rumbo, C. , Tomás, M. , … Bou, G. (2014). Molecular mechanisms involved in the response to desiccation stress and persistence in *Acinetobacter baumannii* . Journal of Proteome Research, 13, 460–476. 10.1021/pr400603f 24299215

[mbo3740-bib-0027] Gebhardt, M. J. , Gallagher, L. A. , Jacobson, R. K. , Usacheva, E. A. , Peterson, L. R. , Zurawski, D. V. , & Shuman, H. A . (2015). Joint transcriptional control of virulence and resistance to antibiotic and environmental stress in *Acinetobacter baumannii* . MBio, 6, e01660–15. 26556274 10.1128/mBio.01660-15 PMC4659468

[mbo3740-bib-0028] Giannouli, M. , Antunes, L. C. , Marchetti, V. , Triassi, M. , Visca, P. , & Zarrilli, R. (2013). Virulence‐related traits of epidemic *Acinetobacter baumannii* strains belonging to the international clonal lineages I‐III and to the emerging genotypes ST25 and ST78. BMC Infectious Diseases, 13, 282 10.1186/1471-2334-13-282 23786621 PMC3691691

[mbo3740-bib-0029] Gruzdev, N. , McClelland, M. , Porwollik, S. , Ofaim, S. , Pinto, R. , & Saldinger‐Sela, S. (2012). Global transcriptional analysis of dehydrated *Salmonella enterica* serovar Typhimurium. Applied and Environmental Microbiology, 78, 7866–7875. 10.1128/AEM.01822-12 22941081 PMC3485933

[mbo3740-bib-0030] Gruzdev, N. , Pinto, R. , & Sela‐Saldinger, S. (2012). Persistence of *Salmonella enterica* during dehydration and subsequent cold storage. Food Microbiology, 32, 415–422. 10.1016/j.fm.2012.08.003 22986208

[mbo3740-bib-0031] Hanczvikkel, A. , & Tóth, A. (2018). Quantitative study about the role of environmental conditions in the survival capability of multidrug‐resistant bacteria. Journal of Infection and Public Health, 10.1016/j.jiph.2018.05.001 29784578

[mbo3740-bib-0032] Jacobs, A. C. , Sayood, K. , Olmsted, S. B. , Blanchard, C. E. , Hinrichs, S. , Russell, D. , & Dunman, P. M. (2012). Characterization of the *Acinetobacter baumannii* growth phase‐dependent and serum responsive transcriptomes. FEMS Immunology and Medical Microbiology, 64, 403–412. 10.1111/j.1574-695X.2011.00926.x 22211672

[mbo3740-bib-0033] Janning, B. , & In't Veld, P. H. (1994). Susceptibility of bacterial strains to desiccation: A simple method to test their stability in microbiological reference materials. Analytica Chimica Acta, 286, 469–476. 10.1016/0003-2670(94)85092-5

[mbo3740-bib-0034] Jawad, A. , Heritage, J. , Snelling, A. M. , Gascoyne‐Binzi, D. M. , & Hawkey, P. M. (1996). Influence of relative humidity and suspending menstrua on survival of *Acinetobacter* spp. on dry surfaces. Journal of Clinical Microbiology, 34, 2881–2887. 8940416 10.1128/jcm.34.12.2881-2887.1996 PMC229427

[mbo3740-bib-0035] Jawad, A. , Seifert, H. , Snelling, A. M. , Heritage, J. , & Hawkey, P. M. (1998). Survival of *Acinetobacter baumannii* on dry surfaces: Comparison of outbreak and sporadic isolates. Journal of Clinical Microbiology, 36, 1938–1941. 9650940 10.1128/jcm.36.7.1938-1941.1998 PMC104956

[mbo3740-bib-0036] Juma, N. A. , Manning, G. , & Forsythe, S. J. (2016). Desiccation survival of *Acinetobacter* spp. in infant formula. Food Control, 68, 162–166. 10.1016/j.foodcont.2016.03.043

[mbo3740-bib-0037] Katoh, H. , Asthana, R. K. , & Ohmori, M. (2004). Gene expression in the Cyanobacterium *Anabaena* sp. PCC7120 under desiccation. Microbial Ecology, 47, 164–174. 10.1007/s00248-003-1043-6 14749909

[mbo3740-bib-0038] Kempf, B. , & Bremer, E. (1998). Uptake and synthesis of compatible solutes as microbial stress responses to high‐osmolality environments. Archives of Microbiology, 170, 319–330. 10.1007/s002030050649 9818351

[mbo3740-bib-0039] Kolter, R. , Siegele, D. A. , & Tormo, A. (1993). The stationary phase of the bacterial life cycle. Annual Review of Microbiology, 47, 855–874. 10.1146/annurev.mi.47.100193.004231 8257118

[mbo3740-bib-0040] Kramer, A. , Schwebke, I. , & Kampf, G. (2006). How long do nosocomial pathogens persist on inanimate surfaces? A systematic review. BMC Infectious Diseases, 6, 130 10.1186/1471-2334-6-130 16914034 PMC1564025

[mbo3740-bib-0041] Leblanc, J. C. , Gonçalves, E. R. , & Mohn, W. W. (2008). Global response to desiccation stress in the soil actinomycete *Rhodococcus jostii* RHA1. Applied and Environmental Microbiology, 74, 2627–2636. 10.1128/AEM.02711-07 18326668 PMC2394902

[mbo3740-bib-0042] Li, H. , Bhaskara, A. , Megalis, C. , & Tortorello, M. L. (2012). Transcriptomic analysis of *Salmonella* desiccation resistance. Foodborne Pathogens and Disease, 9, 1143–1151. 10.1089/fpd.2012.1254 23237410

[mbo3740-bib-0043] Louis, P. , Trüper, H. G. , & Galinski, E. A. (1994). Survival of *Escherichia coli* during drying and storage in the presence of compatible solutes. Applied Microbiology and Biotechnology, 41, 684–688. 10.1007/BF00167285

[mbo3740-bib-0044] Neely, A. N. (2000). A survey of gram‐negative bacteria survival on hospital fabrics and plastics. Journal of Burn Care & Rehabilitation, 21, 523–527. 10.1097/00004630-200021060-00009 11194806

[mbo3740-bib-0045] Nocker, A. , Fernández, P. S. , Montijn, R. , & Schuren, F. (2012). Effect of air drying on bacterial viability: A multiparameter viability assessment. Journal of Microbiological Methods, 90, 86–95. 10.1016/j.mimet.2012.04.015 22575714

[mbo3740-bib-0046] Orsinger‐Jacobsen, S. J. , Patel, S. S. , Vellozzi, E. M. , Gialanella, P. , Nimrichter, L. , Miranda, K. , & Martinez, L. R. (2013). Use of a stainless steel washer platform to study *Acinetobacter baumannii* adhesion and biofilm formation on abiotic surfaces. Microbiology, 159, 2594–2604. 10.1099/mic.0.068825-0 24025603 PMC3853682

[mbo3740-bib-0047] Potts, M. (1994). Desiccation tolerance of prokaryotes. Microbiological Reviews, 58, 755–805. 7854254 10.1128/mr.58.4.755-805.1994 PMC372989

[mbo3740-bib-0048] Potts, M. (2001). Desiccation tolerance: A simple process? Trends in Microbiology, 9, 553–559. 10.1016/S0966-842X(01)02231-4 11825716

[mbo3740-bib-0049] Ramos, J. L. , Gallegos, M. T. , Marqués, S. , Ramos‐González, M. I. , Espinosa‐Urgel, M. , & Segura, A. (2001). Responses of Gram‐negative bacteria to certain environmental stressors. Current Opinion in Microbiology, 4, 166–171. 10.1016/S1369-5274(00)00183-1 11282472

[mbo3740-bib-0050] Reina‐Bueno, M. , Argandoña, M. , Nieto, J. J. , Hidalgo‐García, A. , Iglesias‐Guerra, F. , Delgado, M. J. , & Vargas, C. (2012). Role of trehalose in heat and desiccation tolerance in the soil bacterium *Rhizobium etli* . BMC Microbiology, 12, 207 10.1186/1471-2180-12-207 22985230 PMC3518184

[mbo3740-bib-0051] Roca, I. , Espinal, P. , Vila‐Farrés, X. , & Vila, J. (2012). The *Acinetobacter baumannii* oxymoron: Commensal hospital dweller turned pan‐drug‐resistant menace. Frontiers in Microbiology, 3, 148. 22536199 10.3389/fmicb.2012.00148 PMC3333477

[mbo3740-bib-0052] Roeßler, M. , & Müller, V. (2001). Osmoadaptation in bacteria and archaea: Common principles and differences. Environmental Microbiology, 3, 743–754. 10.1046/j.1462-2920.2001.00252.x 11846768

[mbo3740-bib-0053] Sakamoto, T. , Yoshida, T. , Arima, H. , Hatanaka, Y. , Takani, Y. , & Tamaru, Y. (2009). Accumulation of trehalose in response to desiccation and salt stress in the terrestrial cyanobacterium *Nostoc commune* . Phycological Research, 57, 66–73. 10.1111/j.1440-1835.2008.00522.x

[mbo3740-bib-0054] Soares, N. C. , Cabral, M. P. , Gayoso, C. , Mallo, S. , Rodriguez‐Velo, P. , Fernández‐Moreira, E. , & Bou, G. (2010). Associating growth‐phase‐related changes in the proteome of *Acinetobacter baumannii* with increased resistance to oxidative stress. Journal of Proteome Research, 9, 1951–1964. 10.1021/pr901116r 20108952

[mbo3740-bib-0055] Tschech, A. , & Pfennig, N. (1984). Growth‐yield increase linked to caffeate reduction in *Acetobacterium woodii* . Archives of Microbiology, 137, 163–167. 10.1007/BF00414460

[mbo3740-bib-0056] van den Broek, P. J. , Arends, J. , Bernards, A. T. , de Brauwer, E. , Mascini, E. M. , van der Reijden, T. J. K. , … Dijkshoorn, L. (2006). Epidemiology of multiple *Acinetobacter* outbreaks in The Netherlands during the period 1999‐2001. Clinical Microbiology and Infection, 12, 837–843. 10.1111/j.1469-0691.2006.01510.x 16882288

[mbo3740-bib-0057] Vriezen, J. A. , de Bruijn, F. J. , & Nüsslein, K. (2006). Desiccation responses and survival of *Sinorhizobium meliloti* USDA 1021 in relation to growth phase, temperature, chloride and sulfate availability. Letters in Applied Microbiology, 42, 172–178. 10.1111/j.1472-765X.2005.01808.x 16441384

[mbo3740-bib-0058] Weber, B. S. , Harding, C. M. , & Feldman, M. F. (2016). Pathogenic *Acinetobacter*: From the cell surface to infinity and beyond. Journal of Bacteriology, 198, 880–887. 10.1128/JB.00906-15 PMC4772598 26712938

[mbo3740-bib-0059] Welsh, D. T. (2000). Ecological significance of compatible solute accumulation by micro‐organisms: From single cells to global climate. Fems Microbiology Reviews, 24, 263–290. 10.1111/j.1574-6976.2000.tb00542.x 10841973

[mbo3740-bib-0060] Welsh, D. T. , & Herbert, R. A. (1999). Osmotically induced intracellular trehalose, but not glycine betaine accumulation promotes desiccation tolerance in *Escherichia coli* . Fems Microbiology Letters, 174, 57–63. 10.1111/j.1574-6968.1999.tb13549.x 10234822

[mbo3740-bib-0061] Wendt, C. , Dietze, B. , Dietz, E. , & Ruden, H. (1997). Survival of *Acinetobacter baumannii* on dry surfaces. Journal of Clinical Microbiology, 35, 1394–1397. 9163451 10.1128/jcm.35.6.1394-1397.1997 PMC229756

[mbo3740-bib-0062] World Health Organization . (2017). Priority pathogens list for R&D of new antibiotics. Retrieved from http://www.who.int/mediacentre/news/releases/2017/bacteria-antibiotics-needed/en/

[mbo3740-bib-0063] Zeidler, S. , Hubloher, J. , König, P. , Ngu, N. D. , Scholz, A. , Averhoff, B. , & Müller, V. (2018). Salt induction and activation of MtlD, the key enzyme in the synthesis of the compatible solute mannitol in *Acinetobacter baumannii* . MicrobiologyOpen, e00614 10.1002/mbo3.614 29575790 PMC6291793

[mbo3740-bib-0064] Zeidler, S. , Hubloher, J. , Schabacker, K. , Lamosa, P. , Santos, H. , & Müller, V. (2017). Trehalose, a temperature‐ and salt‐induced solute with implications in pathobiology of *Acinetobacter baumannii* . Environmental Microbiology, 19, 5088–5099. 10.1111/1462-2920.13987 29124876

[mbo3740-bib-0065] Zhang, Q. , & Yan, T. (2012). Correlation of intracellular trehalose concentration with desiccation resistance of soil *Escherichia coli* populations. Applied and Environmental Microbiology, 78, 7407–7413. 10.1128/AEM.01904-12 22885754 PMC3457116

